# Young’s Modulus and Vickers Hardness of the Hydroxyapatite Bioceramics with a Small Amount of the Multi-Walled Carbon Nanotubes

**DOI:** 10.3390/ma15155304

**Published:** 2022-08-01

**Authors:** Maksym Barabashko, Alexander Ponomarev, Anastasiya Rezvanova, Vladimir Kuznetsov, Sergey Moseenkov

**Affiliations:** 1 B. Verkin Institute for Low Temperature Physics and Engineering of the National Academy of Sciences of Ukraine (B. Verkin ILTPE of NASU), 47 Nauky Ave., 61103 Kharkiv, Ukraine; 2 Institute of Strength Physics and Materials Science of Siberian Branch Russian Academy of Sciences (ISPMS SB RAS), 2/4, pr. Akademicheskii, 634055 Tomsk, Russia; ranast@ispms.ru; 3 Boreskov Institute of Catalysis of Siberian Branch Russian Academy of Sciences (SB RAS), 5, pr. Lavrentieva, 630090 Novosibirsk, Russia; vlkuznetsov@gmail.com (V.K.); moseenkov@gmail.com (S.M.)

**Keywords:** bioceramics, hydroxyapatite, multi-walled carbon nanotubes, Young’s modulus, compression strength, Vickers microhardness, plastic and elastic (reversible) works

## Abstract

The Vickers hardness and Young’s modulus of the hydroxyapatite (HA) bioceramics with a small amount of the multi-walled carbon nanotubes (MWCNTs) were studied by using ultramicrotester Shimadzu for dynamic tests DUH-211. Small concentrations of MWCNTs were from 0.05 to 0.5 wt.%. The argon inert atmosphere and vacuum condition were taken for the prevention of the MWCNTs oxidation. The Brunauer–Emmett–Teller (BET) surface area S_BET_ of the HA-MWCNTs composites was determined by thermal adsorption-desorption of nitrogen. It was found that for HA-MWCNTs sintered in the Ar atmosphere, an increase in the concentration of nanotubes up to 0.5 wt.% leads to a decrease in porosity near 3 times in comparison to HA without MWCNTs additives. The small amount of additives of multi-walled carbon nanotubes leads to an increase in hardness of 1.3 times and compression strength of composite and compression strength of composite that is comparable in absolute values with the literature data of enamel hardness (3–5 GPa) and compression strength (95–370 MPa). The absolute values increase close to linearly with the increase of nanotube concentrations. The Young’s modulus of sintered composite slightly changes with the variation of concentrations of nanotubes and close to the enamel (75–100 GPa). The ratio of plastic work to total work and the ratio of elastic (reversible) work to the total work of deformation of composite HA/MWCNTs are practically constant at a studied range of MWCNTs concentration. The additives of the multi-walled carbon nanotubes lead to both an increase in the elasticity index of ~1.5 times and an increase in the resistance to plastic deformation of ~3 times, which improved the tribological performance of the surface. Plastic and elastic (reversible) work slightly changed.

## 1. Introduction

Different biomaterials, such as titanium dioxide, tricalcium phosphate (TCP), bioactive glasses, calcium silicate, hydroxyapatite, polycaprolactone, glassy carbon, zirconia, composites with carbon nanotubes, and other metallic and ceramic materials are used in implants for bone tissue [1,2,3,4,5,6,7,8,9,10,11].

Although mechanical properties of bone scaffold materials have been improved over recent decades, their use in orthopedics is still limited [4]. Glassy carbon has a brittleness and susceptibility to fracture under tensile stress [5].

Titanium is a bioinert metal with good biostability, biocompatibility and long-term durability [6]. At the same time, the organic fluids can lead to the dissolving of the titanium and forming ions that can react with body tissues and appear as a toxic response [7,8,9,10]. Zirconium can be degraded, can have corrosion and also has poor fracture toughness similar to other metals [11].

The applications of hydroxyapatite (HA) [Ca_10_(PO_4_)_6_(OH)_2_] in bone implants are limited due to the brittleness and low fracture toughness of this material [3,4]. At the same time, over recent decades, it has attracted much attention due to biocompatibility, osteoconductivity, bioactivity and its high chemical similarity to natural bone [4,12,13,14].

The improvement of the mechanical properties of HA can be realized by using reinforcing phases, including carbon nanotubes (CNTs). Remarkable properties of CNTs, such as flexibility, low density, high electrical and thermal conductivity, high Young modulus, fracture toughness, and the ability to transfer loads efficiently across an interconnected network that can allow the use of small amounts of CNTs for obtaining the sufficiently better mechanical properties of composites and prevent or delay the initiation and propagation of cracks in the biocomposite [4,15,16,17].

The multi-walled carbon nanotubes (MWCNTs) help in bone repair by accelerating its growth and have good bone-tissue compatibility [18]. Inflammatory reactions and toxic effects in human bone are negligibly small in the case of integration of MWCNTs into the bone [4,19,20]. The partial degradation of CNTs and their oxidation can occur in vivo [21,22]. It was shown that the degradation rates of CNTs correlated with their diameters and layer numbers [22,23]. Nanotubes with larger diameters have a smaller degradation rate. On the other hand, the increase in the diameter of nanotubes leads to a decrease in mechanical properties [24]. Therefore, MWCNTs with a diameter near 18 nm are promising additives to the composites.

In ref. [25], the compression strength of hydroxyapatite composite was increased up to 15 times by adding the multi-walled carbon nanotubes with an average diameter of 18 nm and a concentration of 0.5 wt.%. The 0.3% MWCNT additives in CS/Gel/nHAp composite scaffolds provide better outcomes for porosity, hydrophilicity, and degradation rate than composites with concentrations of MWCNTs above 0.6% [26].

Nanotubes-hydroxyapatite composites have been prepared using various synthesis techniques, such as the sol–gel process [4,27,28], laser surface alloying [29], spark plasma sintering [15], high-precision plasma spraying [30,31], electrophoretic deposition [32], in situ growth of carbon nanotubes reinforced hydroxyapatite coating [33], pressure-less sintering [34], annealing in vacuum [35].

Previously, both a small increase [36,37] and a decrease [37,38] in Young’s modulus of the ceramics with the MWCNT additives were observed. Therefore, the influence of small concentrations (less than 0.5 wt.%) of MWCNTs on the mechanical properties including Young’s modulus of HA is required to be studied.

The aim of this study was dynamic measurements of Vickers hardness and Young’s modulus of the bioceramics based on hydroxyapatite (HA) with a small amount (less than 0.5 wt.%) of the multi-walled carbon nanotubes (MWCNTs). We focus our attention on the sintering of a composite in which an increase in mechanical properties is achieved by using a very small amount of MWCNT additives (less than 0.5 wt.%) for minimizing possible toxic effects. HA as the matrix is needed for obtaining the implant with high osseointegration and biological activity. The improvement of the mechanical properties of HA is possible by varying the amount of the MWCNT additives and sintering procedure regimes. The change of porosity and the unique mechanical properties of MWCNTs are competitive factors that can lead to the non-monotonic dependence of the mechanical properties of the HA-MWCNTs. In addition, the influence of the MWCNTs on the plasticity index and the resistance to plastic deformation that is important for the tribological performance of the surface has not been studied yet. We will study the change in the plastic and elastic (reversible) works with a variation of the concentration of MWCNTs. All these questions are discussed in this study.

## 2. Materials and Methods

### 2.1. Sintering of HAp–CNT Composite

**Scheme 1:**
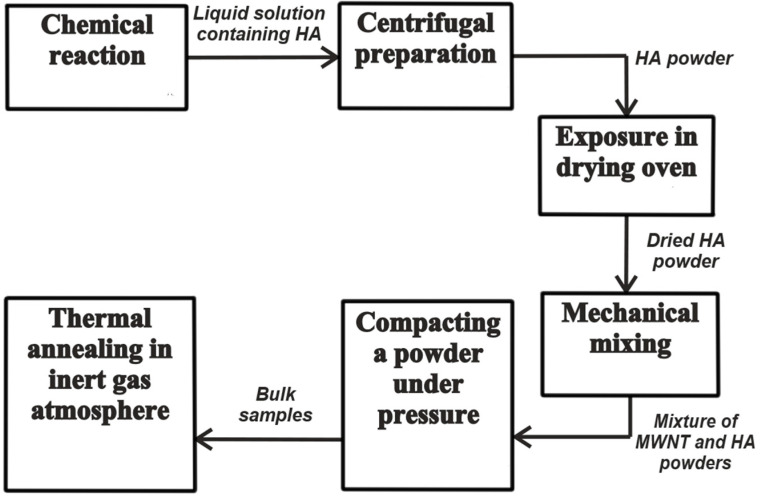
The fabrication process of obtaining the bioactive composite “hydroxyapatite-multi-walled carbon nanotubes” (HA-MWCNTs).

The fabrication process for obtaining the bioactive composite “hydroxyapatite-multi-walled carbon nanotubes” (HA-MWCNTs) was the same as for the samples in ref. [25]. It is schematically shown in Scheme 1.

HA powder was obtained by a wet method using the reaction between CaCO_3_ powder (Merck, Darmstadt, Germany, analytical grade) and an H_3_PO_4_ solution (Merck, Darmstadt, Germany, analytical grade). H_3_PO_4_ solution was rapidly poured into a CaCO_3_ suspension followed by continuous mixing of the reactive medium for 24 h. The mass ratio of reagents was chosen so as to obtain stoichiometric HA [39].

The chemical reaction is described by Equation:

5CaCO_3_ + 3H_3_PO_4_→Ca_5_(PO_4_)_3_OH + 4H_2_O + 5CO_2_ (1)

MWCNTs with low defectiveness and an average diameter of 18 nm were obtained by CVD [40,41]. Concentrations of MWCNTs in the samples of HA (set 1) were 0.05 wt.%, 0.1 wt.%, 0.2 wt.%, 0.3 wt.% and 0.5 wt.%, respectively. Samples were sintered for 1 h in an argon atmosphere at 1100 °C. The increase in the temperature up to 1100 °C was achieved at a constant heating rate of 20 K/min. Partial prevention of the oxidation of MWCNTs was realized by using the argon atmosphere.

Another set of composites was obtained by mixing HA, polyvinyl alcohol (PVOH) and 0.5 wt.%, MWCNTs (set 2). The synthesized HA powder (10 g) was added to a mixture (1 g of PVOH and 10 mL of distilled water). PVOH was added for stabilizing the MWCNTs suspension and in order to increase the overall porosity of the composite, since PVOH evaporates during high-temperature annealing.

The obtained HA-PVOH-MWCNTs were thoroughly treated by ultrasound with a frequency of 35 kHz for 1 h. The resulting powder was dried in an oven for a day at a temperature of 230 °C. The compacted under pressure samples were sintered at a temperature of 1100 °C at vacuum condition 5 × 10^−6^ mm Hg.

### 2.2. Characterization of the Samples

For the characterization of the samples, the X-ray diffraction (X-ray diffractometer Philips, model APDW40C, in copper K_α_ λ = 0.154 nm) and Fourier transform infrared spectroscopy in the mid-IR range (FTIR, BIO-RAD FFS 175 spectrometer, Feldkirchen, Germany, with a resolution of 0.5 cm^−1^) were used. The morphology and microstructure of MWCNTs and the nanocomposites were studied using scanning electron microscopy (SEM, JEOL JSM-7500FA, at 20 kV, Akishima, Japan). The obtained results of the structure of HA and HA-MWCNTs composites agree with the results of the X-ray, IR and SEM investigations in ref. [25]. The apparent porosity and bulk density of the sintered specimens were estimated by Archimedes’ method. The compressive strength was studied previously [25]. The Vickers hardness and Young’s modulus of the polished sintered samples were studied by using ultramicrotester Shimadzu for dynamic tests DUH-211. The samples were polished with diamond paste on a standard metallographic wheel. For each specimen, six indentations were made, and the average was taken as the representative value. The Brunauer–Emmett–Teller (BET) surface area (S_BET_) of the samples was determined by thermal adsorption-desorption of nitrogen in a “Sorbtometr-M” instrument (Katakon, Russia). All the samples were degassed at 120 °C prior to nitrogen adsorption measurements. The BET surface area was determined by a 5-point BET method using the adsorption data in the relative pressure (P/P_0_) range of 0.5–0.8, where P_0_-is atmosphere pressure.

## 3. Results and Discussion

**Figure 1:**
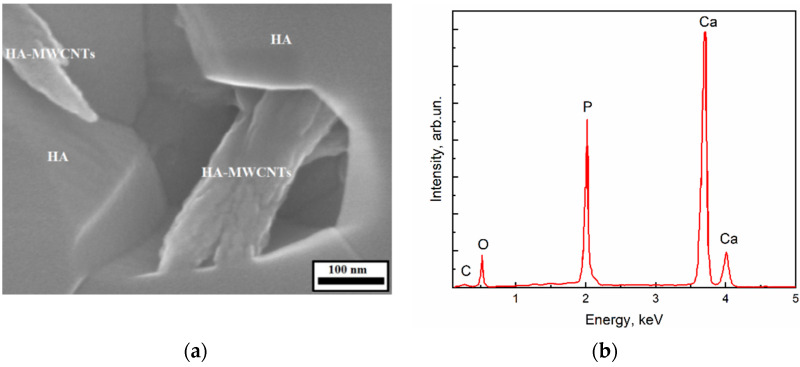

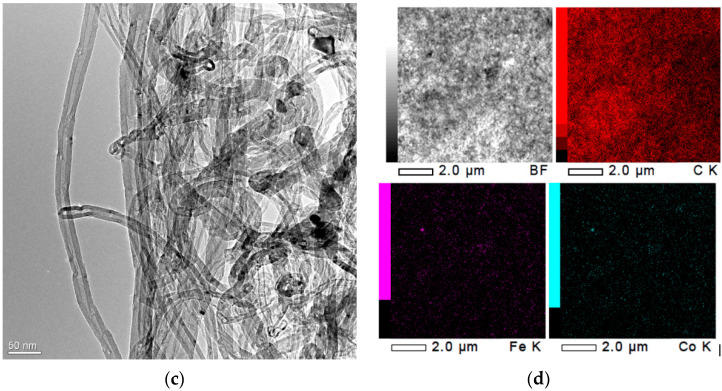
SEM image of hydroxyapatite (HA) composite ceramic with 0.5 wt.% of multi-walled carbon nanotubes (MWCNTs) synthesized at a temperature of 1100 °C in argon atmosphere (**a**) and its energy dispersive X-ray spectroscopy (EDS) spectra (**b**). SEM image of multi-walled carbon nanotubes, (**c**) EDS analysis (SEM image of the cross-section) and elemental mapping of C, Fe, Co (**d**).

The SEM images of the hydroxyapatite (HA) ceramic with 0.5 wt.% multi-walled carbon nanotubes (MWCNTs) sintered in the Ar atmosphere and its energy dispersive X-ray (EDS) spectrum are shown in Figure 1a,b, respectively. From the EDS analysis, the value of Ca/P = 1.71 has been obtained which leads to a smaller density than for the stoichiometric HA (CA/P = 1.67) [42]. It is seen that ropes of several MWCNTs covered by the HA in the composite fill the pores between the HA grains and play the role of “bridge” between different grains, thereby strengthening the composites. Since MWCNTs are the stiffer phase in the composite, their store most of the strain energy [43].

The SEM image of MWCNTs and EDS profile counts of C, Fe, Co are shown in Figure 1c,d, respectively. According to the electron microscope studies, the mean diameter of MWCNTs in our experiment is 18 nm and the metal catalyst particles are encapsulated inside the nanotubes. According to energy dispersive X-ray spectroscopy of the MWCNTs, carbon is the main element. The initial powder of nanotubes contains a small amount of catalyst of Fe (0.17 at.%) and Co (0.09 at.%). The amount of other elements is negligibly small. It means that the sample with the highest concentration of MWCNTs (0.5 wt%) on 1 g of HA has less than 1.5 × 10^−5^ g of particles of the metal catalyst. Therefore, the toxic (Fe/Co) particles in the composite are almost completely absent. In addition, MWCNTs lead to accelerating bone repair, good bone-tissue compatibility [18] and a negligibly small toxic reaction [4,19,20,44]. The degradation rates of MWCNTs decrease with their increasing diameters [22,23].

The fracture toughness of the HA-MWCNTs composite was studied in ref. [45]. It was found that the additives of MWCNTs with a concentration of up to 0.5 wt.% lead to an increase in the fracture toughness of composite ceramics [45]. In ref. [46], it was shown that the MWCNTs lead to an increase in the fracture toughness of ceramics due to their higher strength than the HA matrix, and also due to the possible crack path deflection from the planar geometry and decrease in the crack driving force. MWCNTs are able to transfer loads efficiently across an interconnected network “MWCNTs-matrix” and prevent or delay the initiation and propagation of cracks in the HA-MWCNTs composite [43,47]. The sintering activation due to the presence of MWCNT additives leads to the smaller porosity and as a result, some reduction in the increase in the fracture toughness [46].

HA ceramics have low thermal diffusivity, and the temperature gradients occur between the outer surface and central part of the sample during heterogeneous heating/cooling at sintering [48]. Macrostresses in HA ceramics are higher than in the case of HA ceramics with the additives of MWCNTs. The additives of MWCNTs allow to increase the thermal diffusivity and reduce internal residual macrostresses in the HA-MWCNTs ceramics [48].

The relative porosity of the ceramics HA-MWCNTs was calculated. The porosity of ceramic HA without additives is equal to 27.5%. The porosity of HA with 0.5 wt.% MWCNTs (sintered in Ar atmosphere) is equal to ~8%. The decrease in the porosity of 3.4 times with an increase in the amount of MWCNTs to 0.5 wt.% agrees with the decrease in porosity estimated by using the measurements of the specific surface S_BET_. The ratio between specific surface S_BET_ of pure HA and HA with 0.5 wt.% MWCNTs is equal to ~3. The amount of mesopores with diameters of 2–50 nm is significantly smaller than the accuracy of the study by the nitrogen adsorption with the “Sorbtometr-M” instrument (Katakon, Russia). It is evident that the macrospores make a major contribution to porosity. The decrease in the porosity and the absence of microspores also agrees with the SEM images of the obtained ceramics [25].

From the analysis of the SEM images, it was obtained that in the HA sample the macropores with a size of more than 100 nm make a major contribution to porosity. The obtained composite ceramics HA-MWCNTs have a two-phase structure. It is seen that MWCNTs in the composite fill the pores between the HA grains. The calcium-phosphate matrix is more dense and the macropores are filled by MWCNTs.

The porosity of ceramics HA with concentrations of 0 wt.%, 0.2 wt.%, 0.3 wt.% and 0.5 wt.% MWCNTs (set 1) was 27.5%, 18%, 14.5% and 8%, respectively. It is seen that composite ceramics with additives of MWCNTs have smaller porosity. The results of porosity significantly change at various sintering conditions. The presence of PVOH for the samples of set 2 sintered in a vacuum lead to a porosity similar to the porosity HA without additives.

The next considerations were used in the choice of sintering conditions in our experiment. The sintering conditions (temperature 1100 °C, time of sintering 1 h, relatively high heating rate 20 K/min) were the same for all samples in set 1. According to ref. [42], the bulk density of HA increases with increasing sintering temperature up to 1200 °C. The density of hydroxyapatite is slightly changed with increasing sintering time above 1 h [49].

On the one side, an increase in sintering temperature and sintering time leads to the formation of more dense ceramics. On the other side, the increase of these parameters above chosen leads to an increase in the grain size [50,51], which has a negative effect on mechanical properties.

The fracture toughness of pure HA decreases with increasing temperature above 1000 °C. The highest hardness of HA without additives was achieved in the temperature range 1050–1150 °C [42]. The decomposing of HA into ß-TCP and α-TCP start at a temperature above 1100 °C [42,52].

The variation of the heating rate is also limited. On the one hand, the higher heating rate (40 K/min) can lead to obtaining inhomogeneous ceramic with higher porosity and lower mechanical properties. On the other hand, the lower heating rate (2–6 K/min) eliminates the potential of MWCNTs to improve mechanical properties. Mukherjee et al. [53] obtained the composite of the HA-1 wt.% MWCNTs and HA-2 wt.% MWCNTs with lower Vickers hardness than HA without additives. Composite HA-0.5 wt.% MWCNTs have lower flexural strength than HA without additives. At the same time, the bulk density of the ceramics decreased with an increase in the amount of MWCNTs up to 5 wt.%, which is apparently the main reason for the decrease in their mechanical properties. In the composite HA-MWCNTs, the mass loss is observed at a heating rate of 5 K/min at the temperature range of about 700–1200 °C, which may be due to both dehydroxylation and resulting oxidation of the MWCNTs [54]. The increase in heating rate can minimize the effect of oxidation of nanotubes. The smallest mass loss of MWCNTs was observed at a heating rate in the range of 20–40 K/min [55]. In our study, the argon atmosphere, heating rate of 20 K/min and aligning time of 1 h were chosen as optimal parameters to prevent the oxidation effect of MWCNTs and obtain the HA-MWCNTs with as much as possible higher mechanical properties.

**Figure 2:**
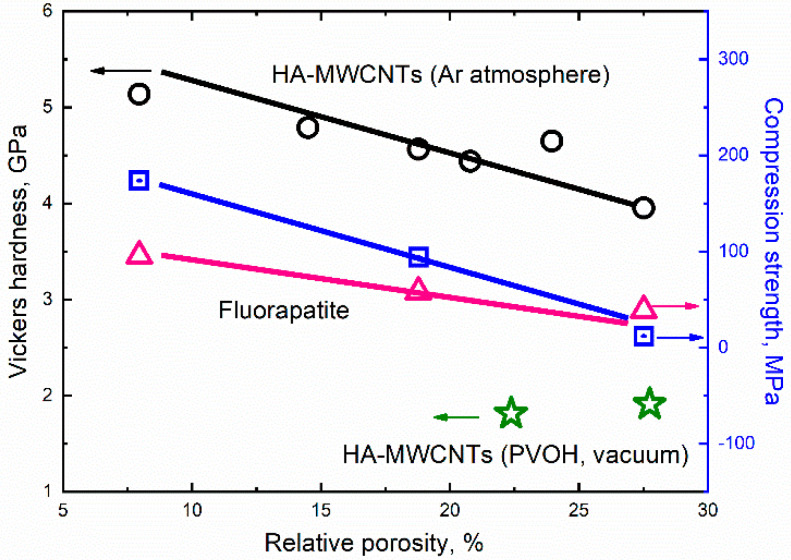
Left axes: Average Vickers hardness vs. relative porosity of the composite HA–MWCNTs sintered in Ar atmosphere, in the range of MWCNTs concentrations from 0 to 0.5 wt.% (open circles) and HA-MWCNTs (0 and 0.5 wt.%MWCNTs mixed with PVOH) sintered in vacuum (open stars). Right axes: Average compression strength [25] vs. relative porosity of the composite HA-MWCNTs (open square) and apatites [49] without MWCNTs additives (open triangles).

The influence of the porosity of materials on the average Vickers hardness and average compressive strength of ceramics HA-MWCNTs (sintered in Ar atmosphere, set 1) and ceramics HA-PVOH-MWCNTs (sintered in a vacuum, set 2) is shown in Figure 2. Multiply coefficient 0.009807 was used to convert Vickers hardness values from HV to Gpa. It is seen that with the increase in the amount of MWCNTs in the samples of set 1, both the Vickers hardness and compressive strength of the ceramics increase. It is seen that the HA with 0.5 wt% MWCNTs has the highest Vickers hardness and compressive strength. The compressive strength of the HA with 0.2 and 0.5 wt% MWCNTs is comparable with the enamel compressive strength (95–370 Mpa) [56].

For set 1, the dependencies of Vickers hardness and compressive strength vs. porosity (P) are well-described by the linear plot with the same angle. It is indicated that the MWCNT additives lead to the formation of more dense ceramics with smaller porosity and higher Vickers hardness and compressive strength. Large porosity in the HA ceramics is apparently due to the higher thermal gradient in the ceramic in comparison with the HA-MWCNTs ceramics [48]. The increase in the porosity due to the presence of a thermal gradient in ceramics has also been observed in refs. [36,57]. The MWCNTs probably decrease the thermal gradients in the ceramics during the sintering due to the better thermal and electrical conductivity of MWCNTs [58,59,60].

Rao et al. [49] described the data of the compressive strength σ of apatites as an exponential dependence of porosity:

σ = 1.38 × 10^8^ × e^−4.6P^, [Pa] (2)

The data of compressive strength σ of apatites [49] is shown in Figure 2 for comparison by triangles. It is seen that our data compressive strength σ of HA–MWCNTs ceramics sintered in the Ar atmosphere (squares) are higher than for apatites without MWCNT additives (triangles). Thus, it is obvious that MWCNTs lead to an increase in hardness and compression strength both due to the intensification of the sintering process and due to the remarkable mechanical properties of the MWCNTs. Both for the compressive strength of the HA-MWCNTs set [25], and for the Vickers hardness of the HA-MWCNTs set (Ar atmosphere), the same slope of the linear curves is observed, which indicates the same trends in the influence of MWCNTs additives on the resulting values of porosity and mechanical properties composites based on HA. The set of composites HA-PVOH and HA-PVOH-0.5 wt.%MWCNTs sintered at the vacuum condition (stars) has smaller values of the Vickers hardness due to the higher porosity. Set 2 was excluded from further analysis due to its low mechanical properties not being promising.

The Vickers hardness (HV) of the obtained bioceramics HA-MWCNTs (set 1) was comparable with the literature data on enamel hardness, which has a value of 5.7 Gpa on the top surface [61] and the lowest value is 3 Gpa of hardness on the “enamel-dentin junction”-EDJ [62]. In ref. [53], the amount of MWCNTs impurity up to 0.5 wt% increases the hardness of the ceramics like in our work. At the same time, we obtained higher absolute values of the hardness of the composite with the same concentration of the MWCNTs, which is apparently due to better sintering conditions in our experiment. In our work, the heating rate of 20 K/min was used to increase the temperature up to 1100 °C. In ref. [53], the temperatures of the samples were increased with a heating rate of 3–6 °C/min up to 1250 °C. An increase in the heating rate decreases the effect of oxidation and decomposition of MWCNT during the annealing [55]. The more optimal chosen temperature is the second argument of the higher Vickers hardness.

**Figure 3:**
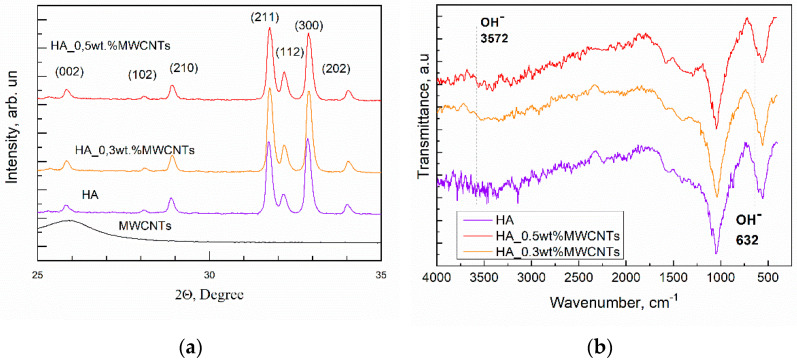
XRD patterns of MWCNTs and composite ceramics HA-0.3 wt.%MWCNTs synthesized at a temperature of 1100 °C in argon atmosphere (**a**). FT-IR spectroscopy of HA-0.3 wt.%MWCNTs ceramics synthesized at a temperature of 1100 °C in argon atmosphere (**b**). Hydroxyapatite (HA) without additives of multi-walled carbon nanotubes (MWCNTs) and HA with 0.5 wt.% MWCNTs are added for comparison [25].

Diffraction patterns of MWCNTs and composite ceramics HA-0.3 wt.%MWCNTs are shown in Figure 3a. Hydroxyapatite (HA) without additives of multi-walled carbon nanotubes (MWCNTs) and HA with 0.5 wt.% MWCNTs were added for comparison [25]. It was shown that all diffraction peaks are indexed to the hexagonal phase of hydroxyapatite with the space group of P63/m and coherent with the Joint Committee on Powder Diffraction Standards (JCPDS-09-0432/1996). The formation of additional phases such as CaO, -TCP, etc., and the broadening of the peaks in the HA matrix were not observed. The crystallite size was near 50 nm [25]. MWCNTs show (002) peak, which is assigned to the hexagonal ring structure of graphite sheets forming carbon nanotubes. The XRD results have coincided with the FT-IR analyses of the ceramics (Figure 3b). The intensity of OH- (632 cm^−1^) vibration modes was weak. It was found from the FT-IR measurements that A-type carbonized apatite is predominantly formed and chosen conditions of sintering. The MWCNTs additives lead to the carbonization of the structure of the hydroxyapatite matrix as the result of partial oxidation of the nanotubes [25].

Lattice parameters (a = b, and c) for the hexagonal structure have been calculated by using the equation [63]:

(3) $$\frac{1}{d^{2}}=\frac{4}{3}\times \frac{h^{2}+\mathrm{hk}+k^{2}}{a^{2}}+\frac{l^{2}}{c^{2}},$$

where d = λ/2 sin θ—lattice distance, h, k, l—Miller’s indexes (the reflection planes).

**Figure 4:**
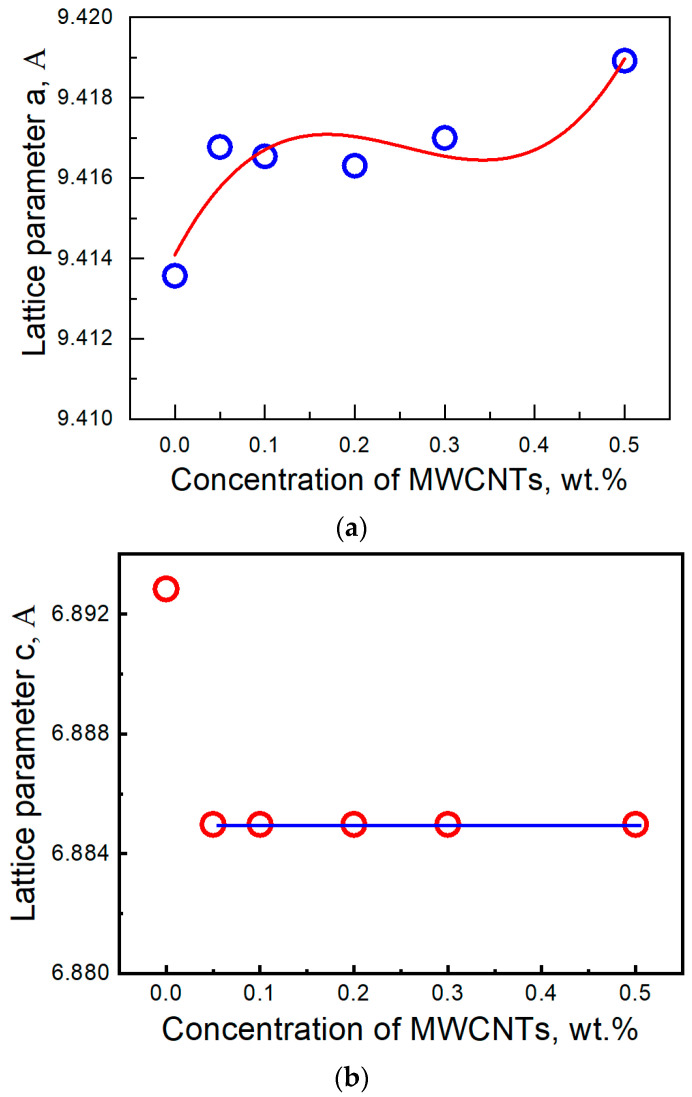
Lattice parameters (a = b, and c) for the hexagonal structure of the HAp composite ceramic vs. MWCNTs amount. Fittings of the experimental points for lattice parameters (a and c) are shown by (**a**) red and (**b**) blue lines, respectively.

It is seen that the lattice parameter a increases (Figure 4a) and lattice parameter c decreases (Figure 4b) with the increase in the MWCNTs additives in comparison with the HA ceramic without MWCNTs. The change of the lattice parameters can be associated with the possible replacement of hydroxyl groups (OH^−^) by carbonate groups as a result of nanotube oxidation. In this case, carbonated hydroxyapatite of A-type is formed.

**Figure 5:**
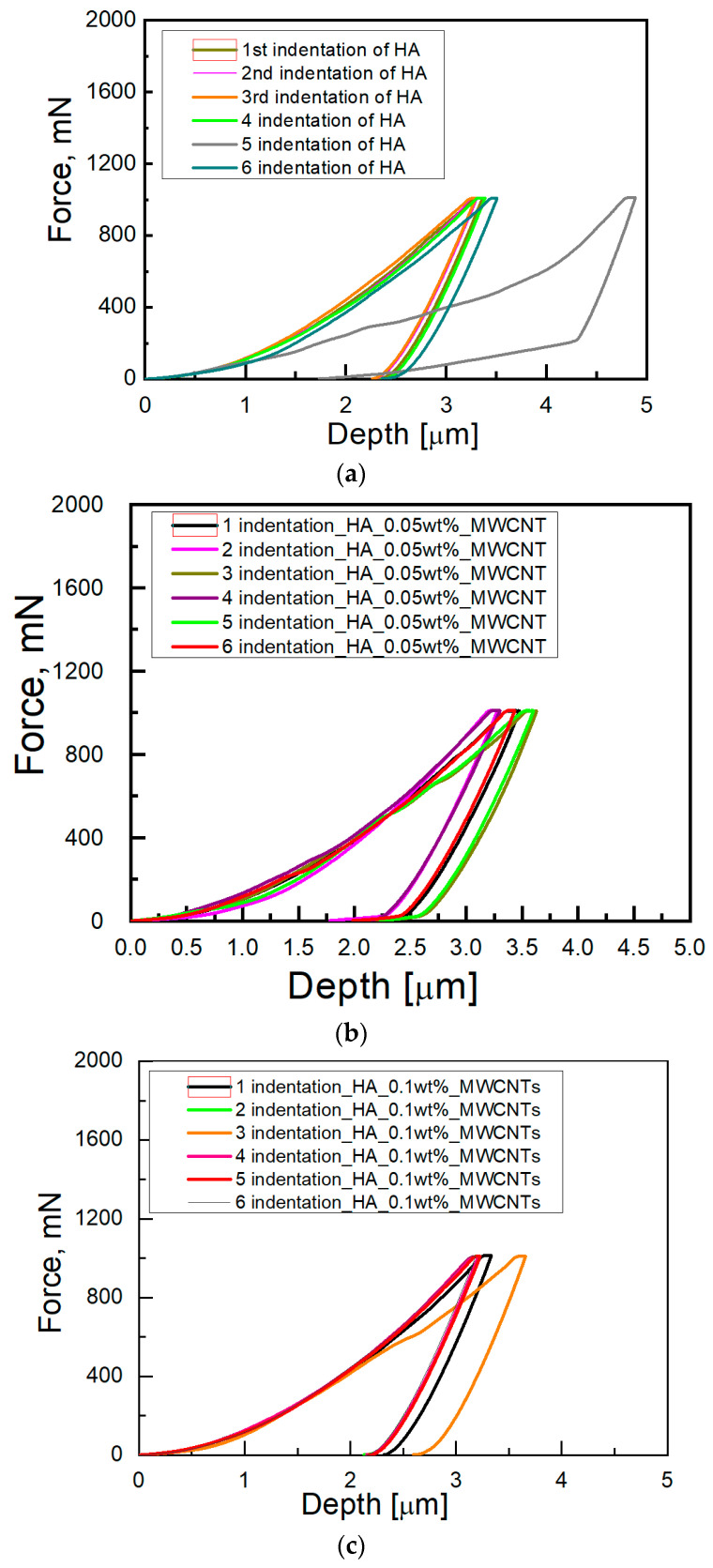

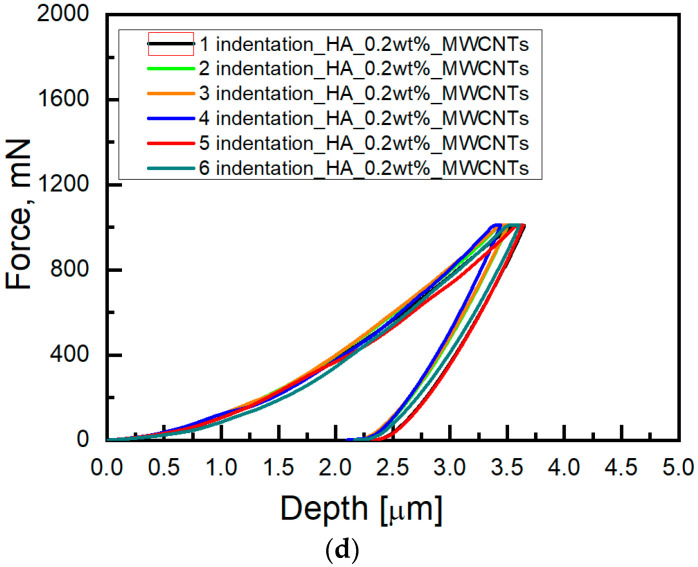
Load vs. displacement plot for HA (**a**) and HA/MWCNTs composites with the amount of MWCNTs additives: 0.05 wt% (**b**), 0.1 wt.% (**c**) and 0.2 wt.% (**d**). Each Figure 5a–d shows the load vs. displacement plots for 6 indentations in the sample.

Measurement of the elastic modulus has been performed on an ultramicrotester Shimadzu by using the indentation technique. The load vs. displacement curves for both HA and HA with MWCNTs are shown in Figure 5a–d. Six indentation tests have been conducted for each specimen.

**Figure 6:**
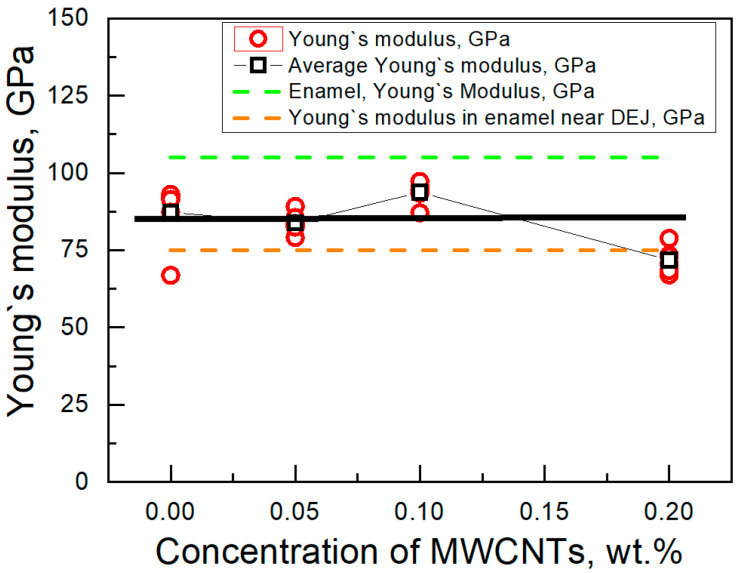
Young’s modulus vs. concentration of MWCNTs. Indentation test (red circles), Average value of the composite HA/MWCNTs (open square). Line shows for the eye the literature values of Young’s modulus for enamel (green line) [61], and Dentin-Enamel Junction (orange line) [64].

The Young’s modulus of the composite vs. concentration of MWCNTs is shown in Figure 6. Red circles show the calculated Young’s modulus for each of the six indentation tests in each specimen. The average Young’s moduli are shown by open square symbols. The black line (average Young’s modulus for the HA ceramics without additives) and green line (the highest and lowers reference value of Young’s modulus for enamel [61,64] are shown for the eye. The significant difference between the Young’s modulus of the sintered composites HA-MWCNTs and the HA ceramics without MWCNTs additives is not observed. At the same time, the Young’s modulus of the sintered composites is comparable with the Young’s modulus of enamel [61]. The decrease in the Young’s modulus of the ceramics may be due to the presence of the MWCNTs with large diameters and the nonhomogeneous distribution of MWCNTs in the samples. The Young’s Modulus of MWCNTs depends on the diameter of the nanotubes and can vary from an E ~1000 GPa (for tubes with a diameter of 7 nm) to E ~10 GPa (diameter more than 40 nm) [24]. The increase of the elastic modulus of HA–MWCNTs composite may be attributed to three major factors: (i) the high Young’s modulus value of MWCNTs with small diameter; (ii) the decrease in the porosity [25,48,64,65] of the HA matrix with the increase in MWCNTs additives and (iii) strong HAp/MWCNTs interface.

The increase in the amount of the MWCNTs additives leads to an increase in the Young’s modulus due to the decrease in the porosity of the HA. On the other side, it was seen in the electron microscope images that the morphology of MWCNTs agglomerates is significantly changed in the ceramics due to partial oxidation of the nanotubes during the sintering [25]. This may lead to a smaller Young’s modulus of the MWCNTs and composite ceramics HA-MWCNTs. Thus, the compensation of factors with the opposite effect in the sample leads to a weak change in the Young’s modulus with an increase in the amount of MWCNTs additives. It agrees with the literature data. Both a small increase [36,37] and a decrease [37,38] in the Young’s modulus of the ceramics with the addition of MWCNTs were observed.

**Figure 7:**
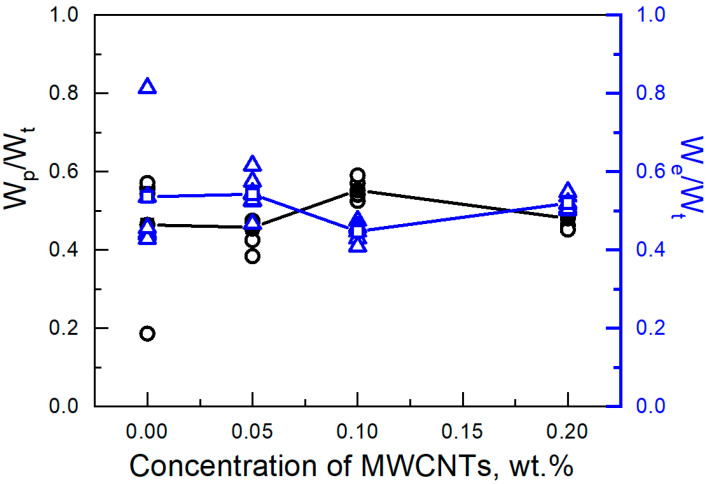
Left axes: Dependence of ratio plastic work to the total work of deformation of composite HA/MWCNTs vs. concentration of MWCNTs (black circle—for indentation tests, and black—square average value). Right axes: Dependence of ratio elastic (reversible) work to the total work of deformation of composite HA/MWCNTs vs. concentration of MWCNTs (blue open triangle—for indentation tests, and blue open square—average value).

Fractions of the plastic (*W_p_*/*W_t_*) and elastic (*W_e_*/*W_t_*) work of deformation during indentation for the composite structure are shown in Figure 7, and were calculated by using the Equations [66]:

(4) $$W_{t}=W_{e}+W_{p}$$

(5) $$\frac{W_{p}}{W_{t}}=\frac{1-3{\left(\frac{h_{f}}{h_{m}}\right)}^{2}+2\;{\left(\frac{h_{f}}{h_{m}}\right)}^{3}}{1-{\left(\frac{h_{f}}{h_{m}}\right)}^{2}}$$

where *W_t_*—total work, *W_p_*—plastic work and *W_e_*—elastic (reversible) work, *h_m_* is the depth of the indent of the peak load and h_f_ is the final depth of indentation after recovery. The obtained values *W_p_*/*W_t_* in such an pproach also agree with the universal relationship between *h_f_*/*h* and *W_p_*/*Wt_ot_* [67]:

(6) $$\frac{w_{p}}{w_{t}}=\left(1+\gamma \right)\frac{h_{f}}{h_{m}}-\gamma \;$$

The values *W_e_*/*W_t_* and *W_p_*/*W_t_* have significant interest in tribology for the prediction of surface deformation based on the work of indentation and vice versa.

It is seen (Figure 7) that the plastic and elastic (reversible) works slightly change with a variation of the concentration of MWCNTs and are approximately equal to half of the total work.

**Figure 8:**
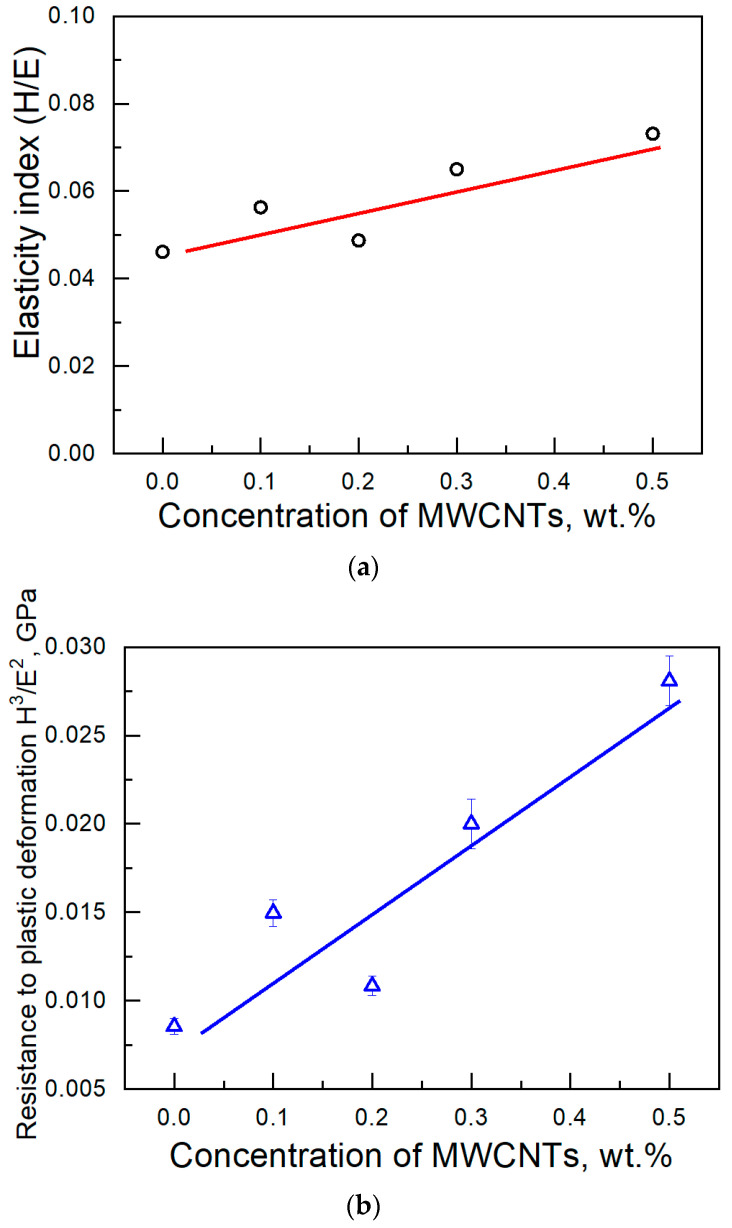
Elasticity index H/E (**a**) and elasticity resistance to plastic deformation H^3^/E^2^ (**b**) vs. concentration of MWCNTs. Linear fittings of the experimental points are shown by lines.

Biomedical components require a protective coating with an optimized balance of hardness and friction [68]. Both H/E (Figure 8a) and H^3^/E^2^ (Figure 8b) increase with the increase in the amount of MWCNTs. The H/E ratio is related to the elastic strain to failure of the surface [69]. The H^3^/E^2^ factor is used to describe the resistance of the material to plastic deformation [70].

It is seen that the additives of the MWCNTs lead to both increase in the elasticity index (H/E) and the resistance (H^3^/E^2^) to plastic deformation. Therefore, the HA-MWCNTs ceramics have a better tribological performance on the surface in comparison with pure HA ceramics for medical application.

## 4. Conclusions

Hydroxyapatite-multi-walled carbon nanotubes (HA-MWCNTs) composite for medical applications were synthesized. The argon inert atmosphere and vacuum condition were taken for the prevention of the MWCNTs oxidation. Concentrations of multi-walled carbon nanotubes were in the range of 0.05–0.5 wt%, an argon atmosphere and temperature of 1100 °C were chosen for sintering. The small amount of additives of multi-walled carbon nanotubes leads to an increase in hardness of 1.3 times. The Vickers hardness values increase close to linearly with the increase in the nanotubes’ concentrations. A similar linear tendency with the variation in the porosity was observed for Vickers hardness and compression strength of the composite. The Young’s modulus of sintered composite slightly changes with the variation of concentrations of nanotubes and close to the enamel (75–100 GPa). The ratio of plastic work to total work and the ratio of elastic (reversible) work to the total work of deformation of composite HA/MWCNTs are practically constant at a studied range of MWCNTs concentration. The additives of the multi-walled carbon nanotubes lead to both an increase in the elasticity index of ~1.5 times and an increase in the resistance to plastic deformation of ~3 times, which improved the tribological performance of the surface. Plastic and elastic (reversible) works slightly changed.

## Data Availability

The data presented in this study are available on request from the corresponding author.
